# Centrally Administered Pertussis Toxin Inhibits Microglia Migration to the Spinal Cord and Prevents Dissemination of Disease in an EAE Mouse Model

**DOI:** 10.1371/journal.pone.0012400

**Published:** 2010-08-25

**Authors:** Jun-xiang Yin, Jiang-long Tu, Hao-jie Lin, Fu-dong Shi, Ru-lan Liu, Chong-bo Zhao, Stephen W. Coons, Sandra Kuniyoshi, Jiong Shi

**Affiliations:** 1 Department of Neurology, Barrow Neurological Institute, Phoenix, Arizona, United States of America; 2 Department of Pathology, Barrow Neurological Institute, Phoenix, Arizona, United States of America; University of California San Francisco, United States of America

## Abstract

**Background:**

Experimental autoimmune encephalomyelitis (EAE) models are important vehicles for studying the effect of infectious elements such as Pertussis toxin (PTx) on disease processes related to acute demyelinating encephalomyelitis (ADEM) or multiple sclerosis (MS). PTx has pleotropic effects on the immune system. This study was designed to investigate the effects of PTx administered intracerebroventricularly (icv) in preventing downstream immune cell infiltration and demyelination of the spinal cord.

**Methods and Findings:**

EAE was induced in C57BL/6 mice with MOG_35–55_. PTx icv at seven days post MOG immunization resulted in mitigation of clinical motor symptoms, minimal T cell infiltration, and the marked absence of axonal loss and demyelination of the spinal cord. Integrity of the blood brain barrier was compromised in the brain whereas spinal cord BBB integrity remained intact. PTx icv markedly increased microglia numbers in the brain preventing their migration to the spinal cord. An *in vitro* transwell study demonstrated that PTx inhibited migration of microglia.

**Conclusion:**

Centrally administered PTx abrogated migration of microglia in EAE mice, limiting the inflammatory cytokine milieu to the brain and prevented dissemination of demyelination. The effects of PTx icv warrants further investigation and provides an attractive template for further study regarding the pleotropic effects of infectious elements such as PTx in the pathogenesis of autoimmune disorders.

## Introduction

Experimental autoimmune encephalomyelitis (EAE) is the primary animal model of multiple sclerosis (MS). The inflammatory reaction in the central nervous system (CNS) is driven by induction of auto-reactive immune cells which survey and penetrate the brain [1], [2]. The vulnerability of spinal cord and the progression of deficits from caudal to rostral in EAE has been attributed to the increased permeability of the blood brain barrier (BBB) and susceptibility of the distal motor and sensory fibers to macrophage/microglia mediated demyelination [3], [4], [5].

There is growing interest regarding the role of pertussis toxin (PTx) on autoimmunity and disease processes such as acute demyelinating encephalomyelitis (ADEM) or MS. PTx is an immune adjuvant utilized to effectively promote inflammation and compromise the BBB facilitating leukocyte infiltration/migration into the CNS [6], [7].

Microglial activation and migration is essential for the development of demyelination and clinical symptoms in EAE [8]. There is considerable evidence of a primary role of microglia in MS as well, particularly secondary progressive MS [9], [10]. Activation of macrophage/microglia dominates the pathologic picture early in the course of disease in EAE, before the onset of clinical symptoms and to a much greater magnitude than T cells [11], [12]. Microglia act as antigen presenting cells (APC) and are responsible for the subsequent activation and infiltration of T cells [8], [13]. Furthermore, they strip and phagocytize myelin stimulating further the induction of auto-reactive CD4 cells [14], [15]. Persistent activation of microglia plays an important role in the development of local inflammatory injury, and the timing of this activation is critical to the progression of autoimmune disease such as EAE.

A time course study in EAE found evidence of two step process mediating T cell and macrophage infiltration [11]. Initially T cells and macrophages access the CNS through the ventricular and meningeal CSF channels. Although there is considerable accumulation of macrophage/microglia and to a lesser extent T cells in the subpial and subependymal regions of the brain, the vasculature appears to be uncompromised. Prior to day 7, there is no evidence of spinal involvement except in the distal meninges of the cord, presumably where the BBB is limited. However, subsequent vascular infiltration and associated demyelination, mediated by macrophage/microglia localize to the distal motor and sensory fibers of the spinal cord [11]. There is considerable evidence that PTx prevents the migration of macrophage/microglia localizing the inflammatory milieu [16]. We hypothesize that confining inflammatory infiltration to the brain through the intraventricular administration of PTx would limit downstream infiltration of the spinal cord. We demonstrate disruption of the BBB and brain parenchymal infiltration of macrophage and to a lesser degree T cells on day 7 in the MOG_33–55_ induced EAE mice after PTx icv. This results in the attenuation of the spinal cord motor symptoms with minimal evidence of infiltration or demyelination. Cytokine concentration and the inflammatory milieu are localized to the brain. There is considerable interest in the basis of pathologic targeting in EAE as well as in MS and its variants [17], [18]. Manipulating pathogen administration in EAE may provide us with clues regarding the role of pathogens in the development of disease as well as in the containment of the monophasic forms.

## Materials and Methods

### EAE induction and treatment

All experimental procedures were approved by the Institutional Animal Care and Use Committee of the Barrow Neurological Institute (Protocol number 309) and performed according to the Revised Guide for the Care and Use of Laboratory Animals. The animals were kept in groups on a 12∶12 h light/dark cycle with food and water ad libitum.

EAE was induced in female C57BL/6 mice (6–8 weeks old, Taconic Laboratory, New York, USA) by subcutaneous injection with 200 µg myelin oligodendrocyte glycoprotein (MOG_35–55_; M-E-V-G-W-Y-R-S-P-F-S-R-V-V-H-L-Y-R-N-G-K, Bio-synthesis Inc. Lewisville, TX), dissolved in an emulsion of 50 µl of complete Freund's adjuvant containing 0.5 mg of heat killed Mycobacterium tuberculosis (CFA, Difco Laboratories, Detroit, MI) and 50 µl of phosphate buffered saline (PBS). On the day of immunization (day 0) and 48 h later (day 2), PTx (List Biological laboratories Inc.) 200 ng in PBS was injected into the mouse tail vein [19]. Neurological functional tests were performed by an examiner blinded to the treatment status of each animal. Functional data were collected on 7 mouse groups (n = 12/group), 3 PTx icv treatment groups (EAE+ PTx icv 1000 ng, 400 ng, and 200 ng), 2 EAE groups (EAE and EAE+ normal saline (NS) icv) and 2 non-EAE control groups (normal +1000 ng PTX icv and CFA +1000 ng PTX icv). Neurological assessments were reported using a five-point standardized rating scale to evaluate motor deficit: 0 no deficit; 1 tail paralysis; 2 unilateral hind limb weakness; 3 incomplete bilateral hind limb paralysis and/or partial forelimb weakness; 4 complete hind limb paralysis and partial forelimb weakness; 5 moribund state or death [20]. Scores were measured daily for 23 days. The onset of disease was calculated by determining the total number of days from MOG_35–55_ immunization to the onset of symptoms in individual animals. Maximal motor scores and motor scores at day 14 and 21 were compared as were onset of disease.

### Stereotactic intracerebroventricular injection

Mice were anaesthetized by injection of a ketamine/xylamine cocktail on day 7 after MOG_35–55_ immunization and mounted in a stereotactic device. A fine hole was drilled through the skull giving access to the surface of the brain 0.7 mm caudal to bregma and 1.0 mm lateral to the sagittal suture. A guarded, 27-gauge 0.5-in needle was stereotactically inserted, targeting the lateral ventricle (3.5 mm depth). A 10.0-µl Hamilton 1700 series gastight syringe was used to inject 2 µl of normal saline, or PTx (500 µg/ml dissolved in normal saline) into the lateral ventricle over a five-minute period.

### Immunohistochemistry

Mice were euthanized at day 7 14 or day 23 post immunization. Terminally anesthetized mice were intracardiacally perfused with saline followed by 4% paraformaldehyde. The spinal cord and brain were embedded in paraffin and cut into serial 6-µm thick coronal slides. Histological evaluation was performed by staining with hematoxylin and eosin (H&E), Luxol fast blue/periodic acid Schiff agent (LFB/PAS), and Bielschowsky silver impregnation to assess inflammation, demyelination, and axonal pathology, respectively.

Histological scores assessing the degree of inflammation, demyelination, and axonal loss in the spinal cord were evaluated using a semi-quantitative system. In brief, the degree of inflammation was assessed by counting the number of cellular infiltrates in the spinal cord. Digital images were collected using an Axoplan microscope (Zeiss, Thornwood, NY) under bright field setting using a 40X objective. Severity of inflammatory cell infiltration on H&E staining was scored using the following scale as described [21]: 0, no inflammation; 1, cellular infiltrates only around blood vessel and meninges; 2, mild cellular infiltrates in parenchyma (1–10/section); 3, moderate cellular infiltrates in parenchyma (11–100/section); and 4, serious cellular infiltrates in parenchyma (>100/section).

Serial sections of paraformaldehyde-fixed spinal cord were stained with Luxol fast blue for myelin and were assessed in a blinded fashion for demyelination using the following scale [22]: 0, normal white matter; 1, rare foci; 2, a few areas of demyelination; 3, confluent perivascular or subpial demyelination; 4, massive perivascular and subpial demyelination involving one half of the spinal cord with presence of cellular infiltrates in the CNS parenchyma; and 5, extensive perivascular and subpial demyelination involving the whole cord section with presence of cellular infiltrates in the CNS parenchyma. Axonal loss was assessed using the following scale [23]: 0, no axonal loss; 1, a few foci of superficial axonal loss which involves less than 25% of the lateral columns; 2, foci of deep axonal loss and that encompasses over 25% of the lateral columns; and 3, diffuse and widespread axonal loss. At least six serial sections of each spinal cord from each mouse were scored and statistically analyzed by ANOVA. Data were presented as Mean ± Standard deviation (SD).

Immunohistochemistry was performed with rabbit polyclonal antibodies against IL-6 (1∶2000, #ab6672, Abcam Inc; Cambridge, MA), and TGF-β (1∶3000, #ab66043, Abcam Inc; Cambridge, MA) to identify crucial pro-inflammatory cytokines; and against ionized calcium binding adaptor molecule 1 (Iba-1, 1∶2500, Wako Chemicals Inc. LA) for microglia [16] and glia fibrillary acidic protein (GFAP, 1∶400, Millipore Corporation, Billerica, MA) for astrocytes. Sections of brain and spinal cord stained with anti-Iba1 allowed quantification of microglia and assessment of its morphology. We performed a morphological analysis of the changes observed and quantified the microglia in sections of cerebral cortex and spinal cord [24].

Th17 cells were identified by double immunostaining for CD4 (1∶1600, Chemicon, Temecula, CA), and IL-17 (1∶3000, rabbit mAb, #ab40663, Abcam Inc., Cambridge, MA) with two fluorescent conjugated secondary antibodies (FITC conjugated and Texas Red conjugated). Immunolabeling was detected by applying the peroxidase-antiperoxidase procedure with 3, 3′-diaminobenzidine (DAB) as cosubstrate [25]. Negative control slides received identical preparations for immunostaining, except that primary antibodies were omitted.

### Western blot protein analysis

Aliquots of equal amount of proteins were loaded onto a 10% SDS-polyacrylamide gel. After gel electrophoresis, blots were subsequently probed with primary antibodies (anti- IL-6, 1∶1000 #ab6672, anti- IL-17, 1∶3000 #ab40663, anti-TGF-β 1∶1000 #ab66043 Abcam Inc; Cambridge, MA). For detection, horseradish peroxidase-conjugated secondary anti-rabbit antibody was used (1∶10,000, #7074, Cell signaling technology; Danvers, MA), followed by enhanced chemiluminescence development (ECL kit, #34077, Thermo Scientific Pierce, Rockford IL).

Normalization of results was ensured by running parallel Western blots with β-actin antibody (1∶25,000 #ab49900, Abcam Inc; Cambridge, MA). The optical density was quantified using an image densitometer (Model GS-670, BioRad, Hercules, CA). The data are presented as a percentage of target protein relative to β-actin. A value of p<0.05 is considered significant.

### BBB studies

Qualitative (immunohistochemistry) and quantitative (Western blot) analyses of exogenous rabbit IgG penetration across the BBB into the CNS were used to evaluate the extent of regional breakdown of the BBB in EAE and EAE+ PTx icv mice [20]. Normal and PTx icv (without EAE) were used as controls. Mice were injected intraperitoneally (i.p.) with 100 µg purified rabbit IgG (Ir-Rb-Gf, Innovative research, Novi, MI, USA) on day 7 (four hours after PTx icv in the EAE+ PTx icv group) or day 14. Animals were euthanized 18–19 hours after the injection. For immunohistochemistry, paraffin embedded sections were probed directly with biotinylated anti-rabbit IgG (1∶100; Vector laboratories, Brulingame, CA). For Western blot, the horseradish peroxidase-labeled anti-rabbit antibody (1∶5000, Cell Signaling Technology, Davers, MA) was used.

### T cell proliferation assays

Animals were sacrificed on day 14. Mononuclear cells were isolated from the spleen and were suspended in culture medium containing DMEM supplemented with 1% penicillin-streptomycin and 10% (v/v) FBS (Invitrogen Life Technologies). Mononuclear cells were then seeded onto 96-well plates at a concentration of 4×10^5^ cells/well. Ten microliters of MOG_35–55_ peptide (10 µg/ml), PLP_139–151_ peptide (10 µg/ml), or Con A (5 µg/ml; Sigma-Aldrich) were then added in triplicate into the wells. After 3 days of incubation, the cells were pulsed for 18 h with 10-µl aliquots containing 1 µCi of [*methyl*-^3^H] thymidine (42 Ci/mmol; Amersham Biosciences). Cells were harvested onto glass fiber filters, and the thymidine incorporation was measured. The results were expressed as Δcpm (DCPM) (mean cpm stimulated cultures – mean cpm unstimulated cultures) [19].

### Flow cytometry analysis

To evaluate the frequency of CD4^+^, CD8^+^, CD4^+^/CD25^+^, CD3^−^/CD19^+^, CD45^+^/CD11b^+^ cells, spleen mononuclear cell culture was prepared from each group on day 14 (the peak of auto-immune response). Single cell suspensions (2×10^6^ cells/5 ml BD tube) were incubated with combinations of fluorescent antibodies, for 30 min at 4°C: CD3 (17A2), CD19 (1D3), CD4 (GK 1.5), CD8 (53–6.7), CD25 PC61.5), CD11b (M1/70), and CD45 (RA3-6B2). The indicated antibodies were fluorescently tagged with either FITC, PE, allophycocyanin, PE-Cy5, PE-Cy7 or APC-Cy7. All purchased from BD Pharmingen. After incubation, each suspension was washed twice (400 g, 5 min, 4°C) with PBS containing 2% bovine serum albumin (BSA) and was resuspended in PBS with 0.5% of paraformaldehyde. Appropriate isotype controls were included. All samples were analyzed on Accuri C6 Flow Cytometer (Accuri Cytometers Inc, USA). Data were analyzed on CFlow Plus software. The number of mononuclear cells per mouse spleen was counted on hemocytometer and the absolute number of a cell subset was calculated based on the percentage of cells stained for the appropriate markers [19].

### Cytokine quantification by Enzyme-Linked Immunosorbent Assay (*ELISA*)

To assess cytokine expression, spleen mononuclear cells were prepared as described above. Suspensions were incubated in RPMI-1640 medium at 37°C for 2 days (2×10^6^ cells/well) with or without antigens (MOG_35–55_ 10 µg/ml or Con A 5 µg/ml, Sigma,USA). Supernatants were collected and aliquoted in 96-well plate precoated with antibodies to Interferon γ (IFN- γ), Tumor Necrosis Factor α (TNF-α), Interleukin-2 (IL-2), Interleukin-4 (IL-4), Interleukin-6 (IL-6) and Interleukin-10 (IL-10) (ELISA Max™ Set Deluxe, BioLegend Inc. San Diego, CA). Optical density was measured at 450 nm on Model 680 Microplate Reader (Bio-Rad Laboratories, Corston,UK). The optical density was quantified by GraphPad Prism 4 (GraphPad Software,Inc) using the standard curve provided by the manufacturer [19].

### Primary microglia cell culture

Cortical tissue was harvested from 0 or 1-day-old C57/BL6 mouse pups (Taconic, Hudson, NY). Meninges and visible vasculature were removed under a dissecting microscope. Cortical tissue was digested in the DMEM/F12 media (Invitrogen Corporation, CA) containing 0.25% trypsin and EDTA (1 mM) at 37°C for 15 minutes. The digested tissue was resuspended in 20 ml media containing DMEM/F12 supplemented with 15% heat inactivated fetal bovine serum, 5% Horse serum (Sigma, St. Louis, MO) and 1% Penicillin-Streptomycin and filtered through a 70-µm nylon mesh (BD Biosciences, San Jose, CA). The cells were washed and seeded in a 75 cm^2^ flask in fresh culture medium (3–4 Pups/per flask). The purity of the microglia cultures was assessed by double-immunostaining with microglial special markers anti Ionized calcium binding adaptor molecule 1 (Iba-1, 1∶2500, Wako Chemicals Inc. LA) and glia fibrillary acidic protein (GFAP, 1∶400, Millipore Corporation, Billerica, MA). The purity of this primary microglia cell culture is about 90–95% [26].

### Microglia migration assay

The migration of microglia *in vitro* was determined by using Transwell (pore size 8-µm, Corning, VWR, San Dimas, CA). Cell-free DMEM/F-12 media (0.8 ml) with or without IFN-γ (20 ng/ml, BD Biosciences, San Jose, CA) was placed in the lower chamber. Microglia suspension (0.1 ml, 5×10^4^ cell/per well) was placed in upper chamber and incubated with or without PTx (100 ng/ml, Campbell, CA) for 24 hours at 37°C. The inserts were then removed and the upper surface was carefully cleansed with cotton pads. Cells on the lower surface were air dried and stained for microglia. Microglial migration was quantified and compared among the groups by counting the number of cells that migrated through the membrane to the lower chamber. Five random fields at 40X fields were counted for each condition under a phase contrast microscope. Each experiment was repeated three times. Results were shown as the cells counted per 40X field [27].

### Statistical analysis

Data were analyzed with SPSS version 10 for windows. The two-way analysis of variance was applied to determine the significance of the difference among the experimental groups. Kruskal-Wallis nonparametric analysis was used for data presented as percentage. The Mann-Whitney U test was used when Kruskal-Wallis showed significance among groups. P<0.05 was considered significant.

## Results

### 1. PTx icv prevents against dissemination of motor deficits in EAE and has a dose effect

**Figure 1:**
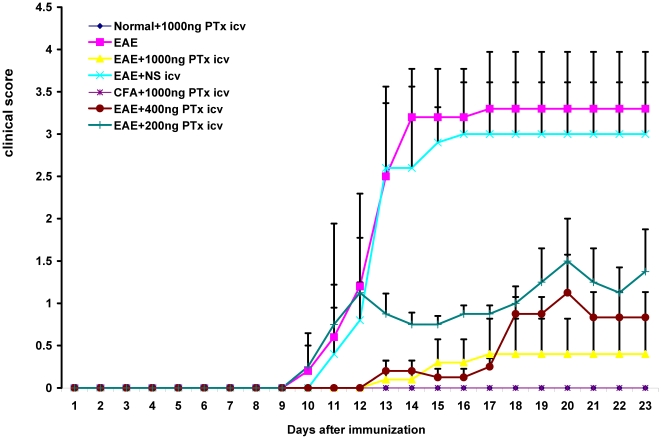
EAE +PTx icv mice developed an attenuated and delayed course of EAE. Clinical scores were evaluated daily in EAE +PTx icv and control mice and were plotted as the mean ± S.D (n = 12/group). Maximum clinical scores as well as scores on day 14 and 23 evidence marked attenuation of disease severity after PTx icv (P<0.01). A dose response to PTx icv is demonstrated as well. Mice receiving lower doses of PTx icv (400 ng and 200 ng) continued to manifest a dose dependent benefit compared to the EAE controls (P<0.05).

PTx icv (1000 ng) delayed the onset of motor symptoms (11.6±0.64 versus 8.5±0.75, p<0.05) and decreased the severity of motor impairment (maximal clinical score 0.35±0.07 vs. 3.25±0.37, p<0.01) (Fig. 1). We evaluated whether there was a dose effect associated with administration of PTx icv (200 ng, 400 ng, and 1000 ng). There was a significant dose effect. The 1000 ng group provided a significantly greater therapeutic response than the 400 ng, and the 400 ng greater than the 200 ng (p<0.05) which also provided a significant therapeutic response relative to EAE (p<0.05) (Fig. 1).

To control for potential effects of icv administration, EAE mice were treated with same volume of normal saline icv (EAE +NS icv). Motor deficits were unchanged compared to EAE alone (Fig. 1). To determine whether the effects of the spinal cord lesion could be alleviated following symptom onset, PTx icv was administered immediately after the onset of measurable motor deficits (clinical score>0.5; day 9+ post MOG_35–55_ inoculation). The delayed administration did not alter the clinical course of EAE (n = 6; data not shown).

### 2. The variation in clinical disease is not due to differences in auto-reactive T cell priming

**Figure 2:**
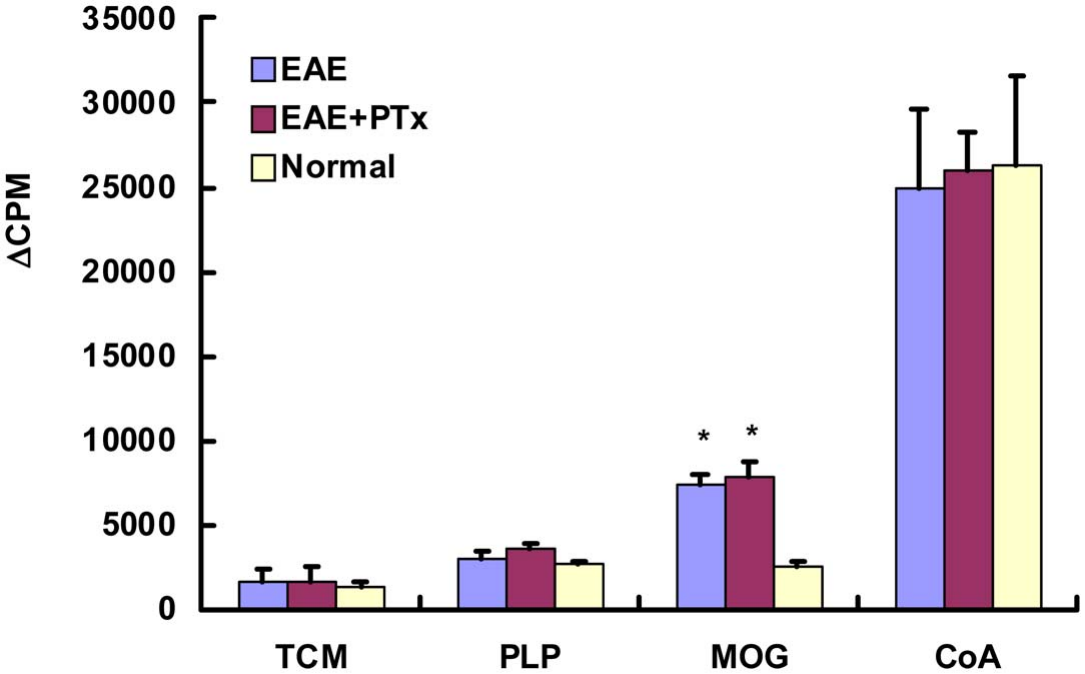
T cell proliferation responses to the Ag (MOG_35–55_ peptide) were assessed in triplicate wells for each experiment. It showed a significant difference in PTx+ EAE and EAE versus control (* *p*<0.01). But there was no difference between PTx+ EAE and EAE mice. Results are expressed as Δcpm (mean cpm stimulated cultures – mean cpm unstimulated cultures). N = 6/group.

**Figure 3:**
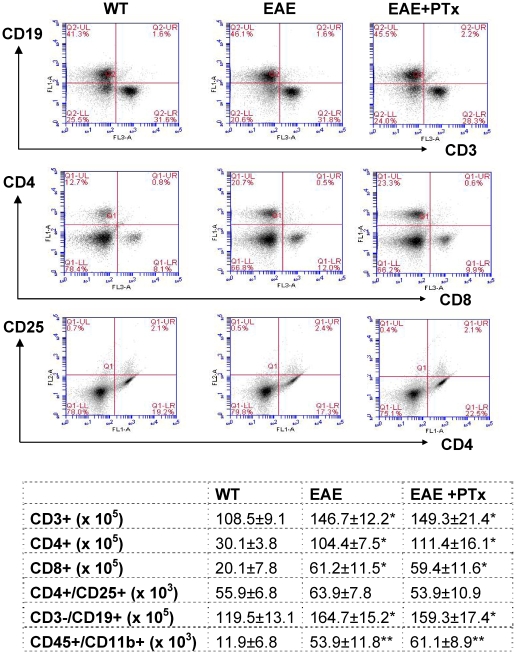
Flow cytometry analysis of mononuclear cells from the spleen on day 14. PTx icv does not alter the peripheral lymphocyte subpopulation in acute EAE. Dot plots of flow cytometry results generated after gating on lymphocytes (by forward and versus side scatter) are shown for T (CD3^+^, CD4^+^, CD8^+^, CD4^+^/CD25^+^ and B (CD3^−^/CD19^+^) cells. WT = wild type group. Absolute numbers of lymphocyte subpopulation, macrophage/microglia cells are shown in the following table. n = 6/group. * p<0.05 compared with WT, * * p<0.01 compared with WT.

To investigate whether an enhanced expansion of auto-reactive T cells could be responsible for the observed clinical differences in EAE versus EAE +PTX icv, T cells were re-challenged with MOG_35–55_ *in vitro*. No differences were observed between EAE and EAE +PTX icv regarding the capacity of T cells to proliferate in response to recall antigen (Fig. 2). Furthermore there was no difference in T cell subpopulations (CD4^+^, CD8^+^, CD4^+^/CD25^+^), B cell (CD3^−^/CD19^+^), and macrophage/microglia (CD45^+^/CD 11b^+^) (Fig. 3). Nor is there a pattern shift in Th1/Th2 between the two groups (Table 1).

**Table 1:** Splenocytes from EAE and EAE+ PTx mice expressed elevated levels of TNF-α, IFN-γ, IL-2, IL-6, and IL-4 compared to WT controls.

pg/ml	WT	EAE	EAE+PTx
**TNF-α**	3.0±0.9	46.7±2.0*	49.3±1.9*
**IFN-γ**	9.9±8.9	2385.9±556.9*	2636.2±186.9*
**IL-2**	5.4±0.6	105.9±26.0*	138.2±23.1*
**IL-6**	14.1±3.8	1144.0±211.5*	1047.0±186.1*
**IL-4**	1.3±0.4	170.9±62.5*	144.3±11.3*

There was no significant difference in cytokine production in EAE and EAE+ PTx. * P<0.001, compared with WT. Abbreviation: WT: wild type, EAE: Experimental autoimmune encephalomyelitis model group, EAE+ PTx: EAE mice with cerebral ventricle injection of Pertussis toxin (PTx).

### 3. PTx icv attenuates spinal cord leukocyte infiltration and demyelination in EAE

**Figure 4:**
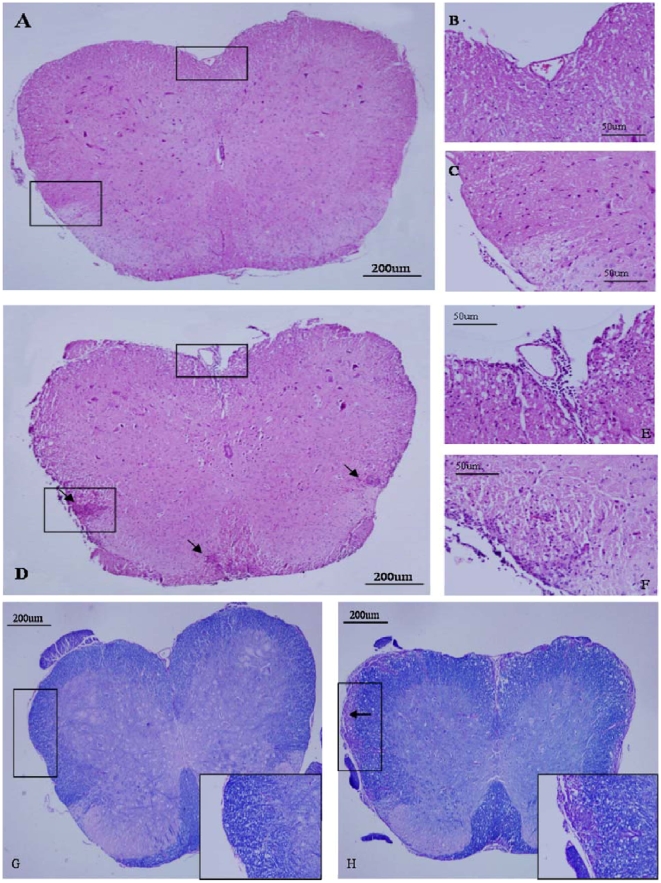
Attenuation of the progression of inflammation and tissue injury in the CNS of mice that received PTx icv. Pathological examination of spinal cord sections from EAE +PTx icv and EAE mice were performed at 7, 14, and 23 days post EAE induction to evaluate CNS inflammation, demyelination and axonal damage. In EAE +PTx icv mice, the number of immune-cell infiltrates (H&E staining, Fig. 4A–C) and demyelination (Luxol fast blue staining, Fig. 4G) were both significantly reduced at day 14 and 23 post EAE induction. Representative day 14 images of H&E staining (A–F) and LFB/PAS staining (G, H). B and C were inserts in A; E and F were inserts in D. Original magnification ×40 in A, D, G and H; ×200 in B, C, E, F, and inserts in G and H.

On day 14 and 23, H&E staining in the cross-sectional of the spinal cord of EAE mice showed widespread infiltration of inflammatory cells in the spinal cord (Fig. 4D–F). In contrast, EAE+PTx icv mice exhibited markedly decreased infiltration of inflammatory cells in the spinal cord on day 14 and 23. (Fig. 4A–C, Table 2).

**Table 2:** Histopathological analyses of inflammatory parameters, demyelination and axonal damage in the spinal cord of C57BL/6 mice at 7, 14, and 23 days after MOG_35–55_ EAE induction.

	EAE	EAE+PTx icv	P value
Inflammation (H&E)			
**day 7**	0.25±0.27	0.08±0.20	0.260
**day14**	3.33±0.75	1.33±0.75	0.001*
**day 23**	3.42±0.58	1.33±0.68	<0.001*
Demyelination (Fast blue)			
**day 7**	0.25±0.27	0.08±0.20	0.260
**day 14**	3.66±0.98	0.83±0.98	0.001*
**day 23**	3.75±0.93	1.16±0.98	0.001*
Axonal loss (silver staining)			
**day 7**	0.83±0.20	0.04±0.10	0.664
**day 14**	2.42±0.86	0.66±0.51	0.002*
**day 23**	2.58±0.97	0.58±0.49	0.001*

Data were presented as Mean ± SD.

To determine the degree of demyelination, we stained sections of spinal cord with Luxol fast blue and observed widespread demyelination zones in the white matter of the spinal cord of EAE mice on day 14 and 23 (Fig. 4H). In contrast, on day 14 and 23, mice that received PTx icv had minimal evidence of demyelination indicated by a markedly attenuated course of disease (Fig. 4G, Table 2). Marked axonal loss characterizes the MOG_35–55_ model of EAE, and this is evident in the spinal sections of the EAE mice assessed with Bielschowsky silver impregnation. Attenuation of axonal injury is evidenced in EAE +PTx icv mice (Table 2).

### 4. PTx icv increases BBB permeability in EAE

**Figure 5:**
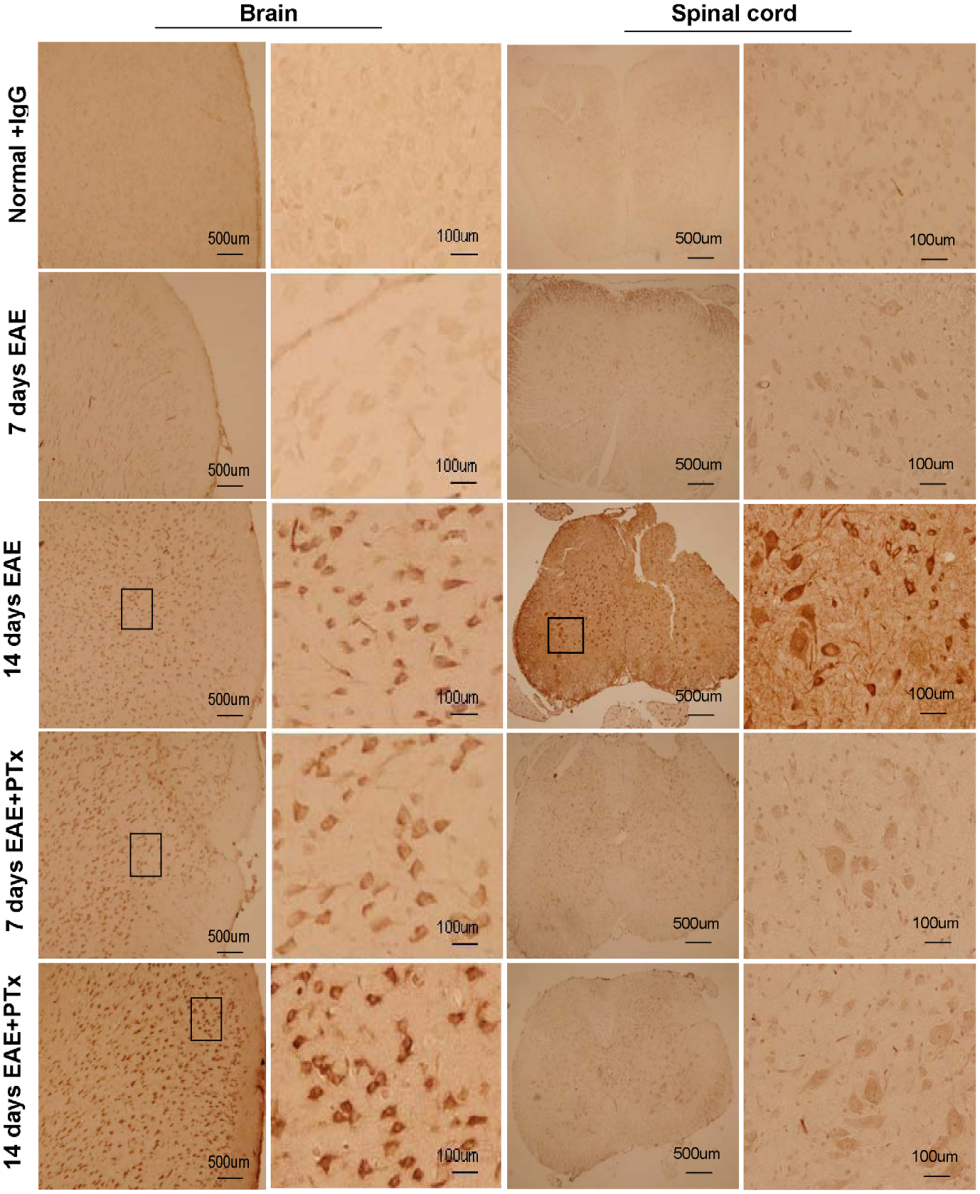
Rabbit immunoglobulin G (IgG) penetration into the frontal lobe parenchyma and thoracolumbar spinal cord in control, EAE, and EAE +PTx icv (n = 7/group). Normal +IgG: age-controlled normal mice without EAE receiving a single i.p. injection of rabbit IgG (100 µg/mouse). 7 days EAE: EAE mice on day 7 post immunization; no penetration of rabbit IgG observed in the brain or spinal cord. 14 days EAE: EAE mice on day 14; marked penetration of rabbit IgG noted in both brain and spinal cord. 7 days EAE +PTx: EAE +PTx icv mice on day 7 post immunization; marked penetration of the brain, but no penetration of the spinal cord. 14 days EAE +PTx: EAE +PTx icv mice on day 14 post immunization, continued evidence of brain penetration, no penetration of the spinal cord. Note the dramatic opening of the BBB on Days 7 in EAE +PTx icv group relative to EAE on day 7.

**Figure 6:**
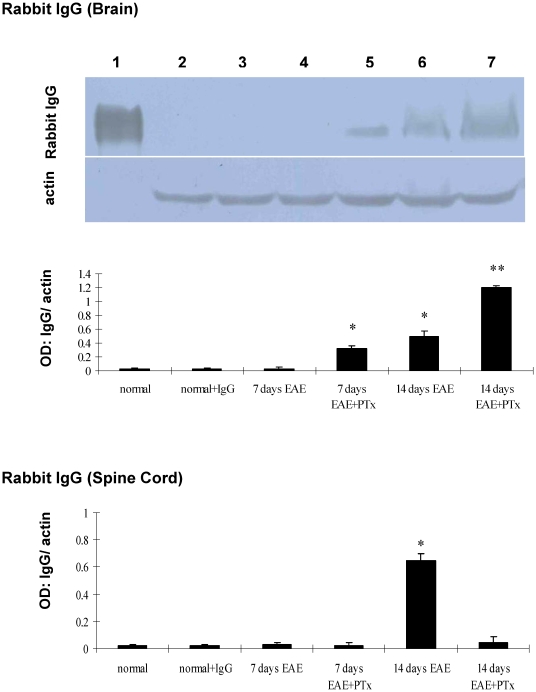
The western blot depicts measures of rabbit IgG. Lane 1: purified rabbit Ig G as the positive control; lane 2–7 correlates the plotted graph below. Statistical evaluation of optic density (OD) normalized to β-actin was obtained for each group. Mean ± SD are depicted (n = 7 per group). *P<0.01, compared with normal control; **P<0.01, compared with normal control group and EAE.

We determined BBB integrity by localizing rabbit IgG in the CNS in EAE and EAE +PTx icv before (day 7) and during the peak (day 14) of symptomatic disease. On day 7, rabbit IgG immunoreactivity was observed in the brains of EAE +PTx icv but not in EAE mice (Fig. 5, 6). In the spinal cord no immunoreactivity was observed in either group. On day 14, EAE mice demonstrated immunoreactivity diffusely throughout the parenchyma of the spinal cord with minimal evidence of reactivity in the brain. EAE +PTx icv mice showed rabbit IgG immunoreactivity in the brain, but not in the spinal cord (Fig. 5, 6).

To control for potential effects of PTx on BBB integrity, separate from its exacerbation of EAE related inflammation, mice were treated with 1000 ng PTx icv but were not exposed to MOG_35–55_. In contrast to EAE +PTx icv mice (Fig. 5, 6), mice that received only PTx icv exhibited no accumulation of rabbit IgG in the brain or the spinal cord (data not shown). Thus, the BBB breakdown described above was caused by the effect of PTx icv in the context of EAE.

### 5. PTx icv preferentially induces the development of myelin-reactive Th-17 cells in the brain

T helper cell lineage development depends on local cytokine milieus and specific immune factors. Emerging evidence supports the pathonogmonic role of Th-17 cells in EAE and the role of PTx in the induction of Th-17 [28]. For the Th-17 cells, TGF-β and IL-6 drive the initial lineage commitment. We quantified the Th-17 cell concentration in our model after PTx icv was administered.

**Figure 7:**
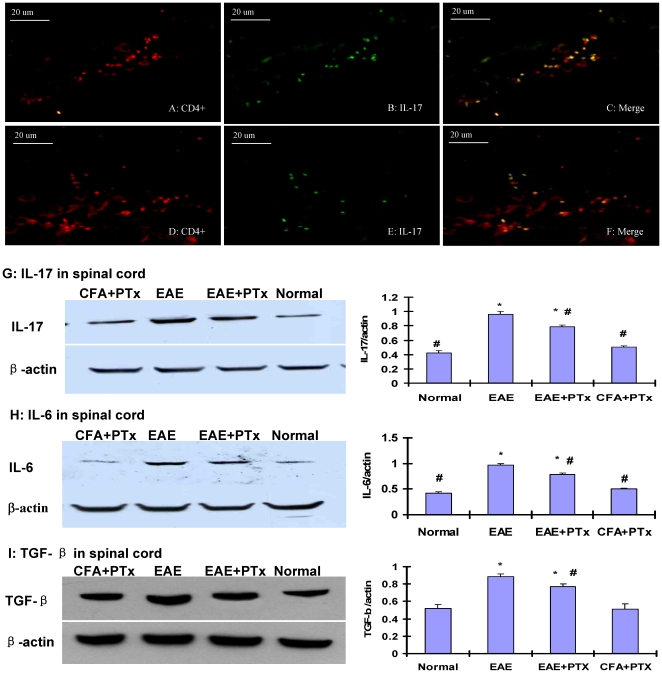
Inflammatory cytokines and cells in the spinal cord of EAE and EAE +PTx icv mice (n = 6/group). IL-17^+^/CD4^+^ cells were detected in the meninges of the spinal cord in the EAE +PTx icv mice (A–C), whereas these cells were diffusely identified in the spinal parenchyma in the EAE mice (D–F). Original magnification ×400. The western blot depicts measures of IL-17 (G), IL-6 (H) and TGF-β (I). In the spinal cord, elevated levels of all three were identified in the EAE mice relative to the EAE +PTx icv mice. Statistical evaluation of optic density (OD) normalized to β-actin was obtained. Mean ± SD are depicted (n = 6 per group). *P<0.05, compared with normal control group; #P<0.05, compared with EAE group.

In the spinal cord, the presence of IL-17 CD4 cells was rare and limited to the meninges in the EAE +PTx icv mice (Fig. 7A–C), whereas a considerable number of Th-17 cells were identified in the spinal parenchyma of the EAE mice (Fig. 7D–F). The protein levels of IL-17, IL6 and TGF-β (Fig. 7G–I) were significantly elevated in the spinal cord of the EAE relative to the EAE +PTx icv mice (p<0.05), correlating the spinal cord pathology in EAE mice.

**Figure 8:**
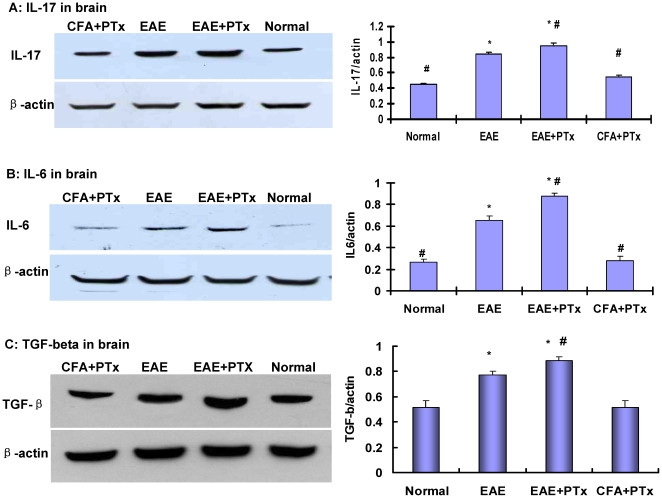
The western blot depicts measures of IL17 (A), IL6 (B) and TGF-β (C) in the brain of EAE +PTx icv compared with in EAE alone mice as well as controls. Statistical evaluation of optic density (OD) normalized to β-actin was obtained. Mean +/− SD are depicted (n = 6 per group). *P<0.05, compared with normal control group; #P<0.05, compared with EAE group.

In the brain, the EAE +PTX icv mice exhibited infiltrating leucocytes which stained positive for CD 4 and IL-17. The majority of these colocalized cells were in the periventricular white matter, confirming the infiltration of proinflammatory of Th-17 cells induced by PTx icv. Whereas, in the EAE alone mice, the presence of Th-17 cells in the brain was limited to the meninges. The protein level of IL-17, IL-6, and TGF-β were significantly elevated in the brain of EAE+ PTx icv mice, relative to the controls and the EAE alone mice (Fig. 8) (p<0.05). In normal control and CFA+ PTx icv groups, no IL-17^+^ cells were detected in brain.

### 6. PTx icv retains macrophage/microglia and to a lesser degree T cell infiltration to the brain preventing dissemination to the spinal cord

**Figure 9:**
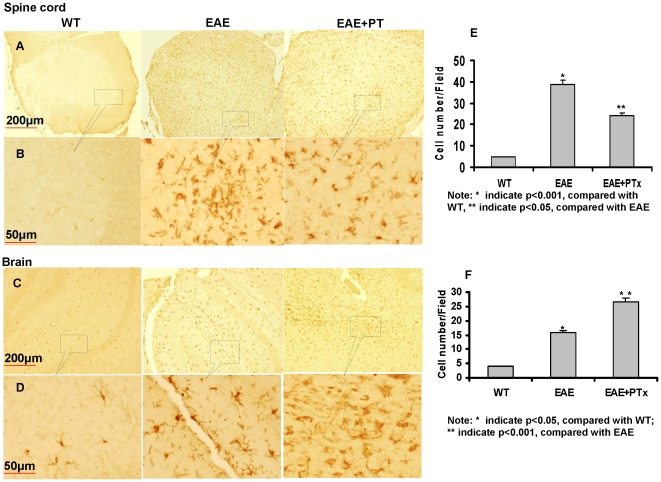
Anti-Iba1 immunostaining of spinal cord and brain of WT, EAE and EAE + PTx icv mice. Brain and spinal cord sections were immuno-stained at 7 days post MOG immunization with the anti-Iba1 antibody. A: Low-magnification image of spinal cord section (Scale bar = 200 µm). The anti-Iba1 antibody reacted strongly with amoeboid-shaped cells, corresponding to activated microglia in the spinal cord of EAE mice. This was significantly less prominent in the EAE+ PTx icv mice. In WT controls, the antibody also effectively, but rather weakly, recognized ramified or resting microglia; these cells have small bodies and finely branched processes. B: High-magnification image of the spinal cord sections (Scale bar = 50 µm). C: Low-magnification image of cerebral cortex (Scale bar = 200 µm). The anti-Iba1 antibody reacted strongly with amoeboid-shaped cells, corresponding to activated microglia in the brain of EAE+ PTx icv mice. WT controls manifest ramified or resting microglia; whereas EAE mice manifest an intermediate stage. D: High-magnification image of the brain sections (Scale bar = 50 µm). E-F. Microglia were quantified and compared among the groups by counting the number of cells in the field. Five random fields at 40X fields were counted for each condition under a digital axoplan microscope. Results were shown as the cells counted per 40X field. * p<0.05 compared with wt; * * p<0.01, Compared with EAE.

**Figure 10:**
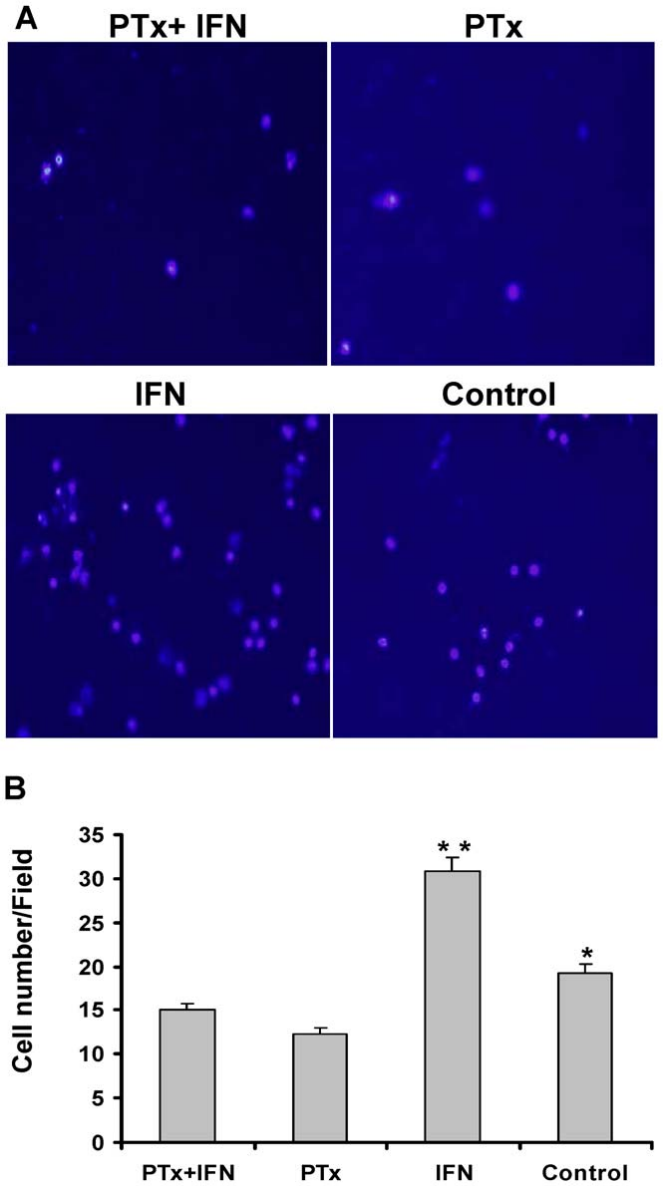
PTx significantly reduced migration of stimulated microglia. Microglia migration was quantified and compared among the groups by counting the number of cells that migrated through the membrane to the lower chamber. Five random fields at 40X fields were counted for each condition under a phase contrast microscope. Results were shown as the cells counted per 40X field (A and B). In PTx treated groups, cell migration was significantly reduced. * p<0.05 compared with PTx; * * p<0.01, Compared with PTx+ IFN, PTx and Control groups.

The most salient finding of PTx icv on day 7 post immunization was the parenchymal infiltration of macrophage/microphage (Iba1), and to a lesser magnitude T cell (CD4), in the brain (Fig. 9). In the brain of EAE+ PTx icv mice, anti-Iba1 antibody reacted strongly with amoeboid-shaped cells, corresponding to activated microglia on day 7. Wild type controls manifest ramified or resting microglia; whereas EAE mice manifest intermediate responsiveness and ramification (Fig. 9-C, D, F). In contrast, the spinal cord of EAE mice showed amoeboid-shaped cells that stained strongly with anti-Iba1 antibody, corresponding to activated microglia (Fig. 9-A, B, E). To further determine the effect of PTx on microglia migration, we utilized the Transwell to assess *in vitro* migration. PTx significantly inhibited the migration of microglia with and without IFN-γ stimulation (Fig. 10).

## Discussion

Several studies in different animal models have demonstrated that macrophage/microglia activation and infiltration are essential for the development of clinical EAE [29], [30], [31]. This has been verified in numerous studies which traced the therapeutic effects to the disruption of macrophage/microglia function [15], [32], [33]. Macrophage cells differentiate into both microglia-like and dendritic-like cells in the CNS during EAE [34]. They function as antigen presentation cells to the encephalitogenic T cells [13], [35]. Macrophage/microglia strip off myelin from axons and through receptor mediated pathways phagocytize myelin [36], [37]. Activated microglia through the production of a proinflammatory milieu disrupt the blood-brain barrier (BBB) integrity, attract and activate of T cells and monocytes which augments the destruction of myelin [33].

Time course studies on MOG induced EAE indicate that immune cell initiation of EAE occurs in stages and inflammatory interactions among resident microglia, invading T lymphocytes and neuronal elements occur prior to the onset clinical EAE in lawful temporal and spatial relationship to one another [11]. Their emergence before the onset of EAE clinical symptoms and their localization in the inflammatory lesions indicate that they play a major role in the initiation and progression of EAE [38].

The earliest stage of EAE, appears to occur on and prior to day 7 (prior to the onset of clinical features), is characterized by a marked accumulation of activated macrophage/microglia in the sub-ependymal (periventricular) and subpial regions of the brain parenchyma. The presence of limited infiltration in these regions and the sparing of vascular structures indicates this stage involves modest infiltration primarily through CSF venues. Furthermore, T cell presence considered primary in the development of EAE is modest compared with the accumulation of macrophage/microglia suggesting a greater T cell stimulatory role of microglia possibly involving their APC potential [11]. This is supported by evidence that subsequent stages (> = 10–14 d) are distinguished by more pronounced infiltration of T cells which define inflammatory foci as well as the onset of motor symptoms [11], [38].

The findings described above in our studies and others indicate that the initial APC and T-cell recruitment in EAE occurs at the meninges and choroid plexus rather than at the vasculature and suggested that the inflammatory responses in the form of microglial activation at distal levels of afflicted pathways might be involved in subsequent macrophage and T cell transit across the vasculature in the spinal cord [11]. After PTx icv however, rapid disruption of the BBB in the brain was detected indicating macrophage and T cell transit across vasculature. PTx icv early in the course of disease prevented downstream disruption of the BBB, whereas later administration (> = 10–14 days) did not alter the course of disease in the spinal cord. This indicates a time dependent cascade mechanism regarding prevention of downstream vasculature compromise.

PTx has a multitude of effects on the immune system. In EAE, the animal model for MS, it has been considered an immune adjuvant responsible for a more severe course of disease and the induction of disease in animals not previously susceptible [39]. PTx degrades of integrity of the BBB, activates microglia, and induces the infiltration of effector T cells and macrophages creating a proinflammatory milieu of cytokines [28], [39], [40]. However, there is considerable evidence that PTx through its direct effect on T cells and antigen presenting cells (APC) inhibits the migration of immune cells preventing dissemination of inflammation [41].

In this study we demonstrate that PTx icv prevents the dissemination of disease into the spinal cord. PTx icv administered on day 7 inhibits the migration of macrophage/microglia mitigating further infiltration and evidence of inflammation and demyelination distally. Our studies confirm evidence of microglial infiltration in the brain early in the course of disease (day 7), in both the EAE and EAE +PTX icv model. However, in the EAE model the infiltration is limited in magnitude and location to the subpial and subependymal regions of the brain, whereas in the EAE +PTX icv model the microglial are prolific throughout the brain parenchyma. It has been hypothesized that downstream accumulation of macrophage/microglia stimulated by Wallerian degeneration which begins rostrally and is responsible for the distal vasculature mediated infiltration and ascending paralysis. Our data suggests that arrest of microglia migration rostrally prevents degeneration and spinal cord infiltration distally by retaining microglia locally abrogating dissemination to the spinal cord.

Our study applied PTx icv to an *in vivo* model to determine the effects of early icv administration of PTx on microglia migration, BBB integrity, lymphocyte infiltration, and subsequent demyelination and dissemination of the disease. Our PTx+EAE model reflects some key features of multiple sclerosis lesions and provides a novel approach to study the mechanism responsible for the localization of demyelinating lesion in the brain versus spinal cord. It may also provide a model to investigate the variable nature of lesions in diseases such as relapsing remitting MS and indicate why the development of lesions in one region of the neuroaxis may temporarily preclude the lesions elsewhere. Finally this model encourages the investigation of the pleotropic effects of infectious elements such as PTx in the pathogenesis of MS and other autoimmune disease.
